# Robotic tracheal resections on veno-venous extracorporeal membrane oxygenation with 23-hour length of stay and without guardian chin stitch

**DOI:** 10.3389/fsurg.2026.1719816

**Published:** 2026-02-03

**Authors:** Ashley J. McCormack, Stephanie H. Chang, Deane E. Smith, Travis C. Geraci, Katherine G. Phillips, Robert J. Cerfolio

**Affiliations:** Department of Cardiothoracic Surgery, NYU Langone Health, New York, NY, United States

**Keywords:** outcome, robot—assisted thoracic surgery, tracheal resection, tracheal tumor, VV ECMO

## Abstract

**Objective:**

Mid-to-distal tracheal surgery for cancer can be safely performed minimally invasively with a one-day length of stay, avoiding a guardian chin suture, and ensuring a R0 resection in select patients.

**Methods:**

This is a retrospective technical review of the largest series to date of patients with mid-to-distal tracheal cancers. All were offered a right robotic approach using veno-venous extracorporeal membrane oxygenation (VV ECMO) support via percutaneous right internal jugular vein and right common femoral vein access.

**Results:**

From May 2019 to April 2024, five consecutive patients (3 men, 2 women; aged 11, 29, 37, 40, and 74 years) presented with a mid-to-distal tracheal cancer. All underwent right robotic mid-distal tracheal resections on VV ECMO for primary tracheal cancers. All patients had an end-to-end tracheal anastomosis and R0 resection and all avoided: systemic heparinization, suprahyoid release maneuvers and a postoperative guardian chin stitch. Median operative time was 258 min (range 227–292). All patients tolerated the operations well and were discharged home on the morning of postoperative day 1. There was no minor or major morbidity, no 30 or 90-day mortality, and no re-admissions. Two patients complained of cough. All had R0 resections and to date none have evidence of recurrent disease or stricture.

**Conclusion:**

Resection of mid-to-distal primary tracheal cancers can be performed safely and efficiently via a right robotic approach while on VV ECMO with little to no morbidity or mortality and require only an overnight hospital stay. The techniques used to perform the operation and achieve these results are described.

## Introduction

Primary tracheal tumors and/or cancers are rare, with an annual incidence of 0.1 per 100,000 per year (1, 2). The management should deliver complete surgical resection when feasible with negative (R0) margins. The surgical approach is dictated by the location of the tumor within the trachea. Tumors in the proximal half can often be approached via a collar incision whereas those in the mid or lower half are traditionally approached via a right thoracotomy (3) in the past and now via a minimally invasive approach. We have advocated and employed the benefits of minimally invasive approaches over thoracotomy to reduce morbidity, muscle trauma, pain, and length of stay, while improving patient experience (4, 5). Cross-table ventilation during distal tracheal resections is cumbersome and risks tearing the airway, especially the left mainstem bronchus. As a solution, we first proposed and offered the value of placing the patient on veno-venous extracorporeal membrane oxygenation (VV ECMO) support in 2019, which not only fully supports the patient's respiratory function (6), but also may make the surgery technically easier. In this series, we present our experience with this first reported technique in five patients via a right-sided robotic approach for primary tracheal cancer while on VV ECMO.

## Patients and methods

### Study design

This is a retrospective review of prospectively gathered data at a single institution from a single thoracic surgeon (RJC). All patients who required a mid-to-distal tracheal resection underwent right robotic tracheal resection on VV ECMO, as described below. There were no patients during this time that had any other approach offered. Primary outcomes included minor and major morbidity, 30- and 90-day mortality, readmission within 30 days, the development of a stricture, and recurrence of cancer. The study design, including a waiver of patient consent, was approved by the Institutional Review Board at NYU Langone Health, i24-01162.

### Perioperative management

All patients who presented with tumors or non-dilatable strictures in the mid or distal trachea were eligible for this operative approach. Patients were evaluated, as we have previously described, by bronchoscopy, computed tomography scan, integrated positron emission tomography, pulmonary function testing and stress test in select patients (7).

### Operative technique

A single lumen endotracheal tube is placed and guided by bronchoscopy by the surgeon to remain proximal to the lesion to avoid bleeding in the airway. We prefer an 8.0 or 8.5 single lumen tube so an adult bronchoscope can be easily placed. A double lumen tube is not required for this operation. The patient is placed in the supine position and placed on VV ECMO via percutaneous access of the right internal jugular vein and the right common femoral vein. No systemic heparin is administered. After the cannulas are secured into place, the patient is carefully positioned in the left lateral decubitus position and ECMO is started. The single lumen tube is pulled back to the upper trachea with the cuff below the vocal cords and it is disconnected from the ventilator. Standard robotic ports are then placed in the right chest with the da Vinci Xi Surgical System (Intuitive Surgical, Sunnyvale, CA) through a portal four-arm approach and an assist port going over the 7th rib instead of the more traditional 9th rib as used for pulmonary resections (4).

Air seal and low pressures of 8 mmHg (instead of the more traditional 10 or 12 mmHg) are used to reduce the CO_2_ sweep required by the VV ECMO circuit. The chest is inspected and the inferior pulmonary ligament is taken down, the mediastinal pleura is incised around the lung and the N2 lymph nodes from stations 9, 8, 7, 2R and 4R are removed. Dissection is performed using bipolar energy to mobilize the trachea and to ensure preservation of the posterolateral blood supply of the trachea in the areas that do not require resection (Supplementary Video S1). The trachea can be fully mobilized with a right robotic approach in the mediastinoscopy plane superiorly (anterior to the trachea, under and superior to the innominate artery) and into the neck. We no longer perform mediastinoscopy as we used to in the past prior to right thoracotomy and thus can now avoid the neck incision.

The proximal and distal airway must also be freed to avoid anastomotic tension. The distal airway is mobilized in several ways. The entire subcarinal lymph node packet must be removed, and the left mainstem bronchus visualized (Supplementary Video S2). Importantly, the posterior aspect of the right upper and the right middle lobe and the right lower lobe airway are freed as well. Supra-hyroid and pericardial release maneuvers are only performed if after full and proper dissection of the airway there remains significant tension on the anastomosis. Both maneuvers add morbidity and should be avoided if possible.

Intraoperative bronchoscopy is used with the tile pro feature and the infrared robotic camera (intravenous ICG is not needed) to help identify the exact proximal and distal extents of the tumor and mark the precise location for the proximal and distal openings in the airway (Supplementary Video S3). If these adjuncts are not available, a small needle can be placed in the airway during bronchoscopy as well to help guide the optimal location for the tracheal opening.

The distal and proximal tracheotomies are performed next (Supplementary Video S4). Prior to dividing the airway, it is critical that the airway is fully dissected free and mobilized as it is more difficult to do after division. Since the patient is off the ventilator there is no oxygen in the system, but it is important that the oxygen concentration is turned down to room air as we routinely do for sleeve resections and thus the risk of an airway fire is mitigated as the airway is opened. Parts of the proximal and distal complete circular tracheal rings are sent for frozen pathology as margins. We favor sending four from each margin so that the pathologist looks at multiple areas, for a total of eight frozen sections at the 12, 3, 6 and 9 o'clock locations of both the proximal and distal margins.

While waiting for the frozen pathology result, we start the anastomosis after first assessing it for tension by trying to bring the two ends together. This can be done by simply holding each end with a robotic instrument and assessing the tension via visual clues. In our experience, this is all that is needed. Another method is to place a suture at the 12 o'clock positions and bring the two ends together, again assessing the degree of tension. If the visual clues are not sufficient (they should be), one can ask the bedside assistant to bring the two ends together or the surgeon can scrub in and bring the two ends together to assess the tension themself. We have not found this step to be necessary.

A tension-free, end-to-end anastomosis between two well-perfused cancer-free ends is performed using a running 3-0 barbed suture, Stratafix (Ethicon), with the knots on the outside of the airway (Supplementary Video S5). We start with the needle going from out-to-in on the distal trachea at the 9 o'clock position that contains tracheal cartilaginous rings and then the needle is placed in-to-out on the proximal 3 o'clock location and run anterior to posterior. Once the cartilaginous portion of the trachea is sewn together, a second suture is run posteriorly to sew the membranous portion. We do not telescope one end into the other. We then perform an underwater leak test after completion of the anastomosis by reattaching the single lumen tube to the ventilator, turning off the CO_2_ insufflation and submerging the anastomosis under saline to ensure there are no bubbles as the right lung inflates (Supplementary Video S6). If not, both lungs remain ventilated.

One 20-Fr soft chest tube is placed and connected to a digital drainage system, Thoraguard (Centese, Omaha, Nebraska) and is assessed. If the air leak is 20 mL/min or less, it is removed before the patient is turned on their side and the chest tube site can be closed with a knotless subcuticular closure. After closing the robotic incisions, the patient is placed in the supine position while protecting the ECMO cannulas. The patient is decannulated from VV ECMO as the lungs are ventilated. After holding direct pressure for ten minutes, these small incisions are also closed with subcuticular knotless sutures. No systemic heparin is administered while on VV ECMO. If the chest tube was not removed while the patient was on their side because the air leak was greater than 20 mL/min at the time of chest closure, the chest tube is then re-assessed for an air leak and removed prior to leaving the OR. No guardian chin stitch is placed. Patients are extubated in the OR and brought to the recovery room.

### Postoperative management

Upon arrival in the recovery room, a portable CXR is obtained. After a brief stay in the recovery room, all patients are transferred to a regular postsurgical floor, not the intensive care unit. The chest tube, if still in, is assessed continuously with plans to remove it within 4–6 h postoperatively, as we have previously described (8). The chest tube is removed if: (1) chest tube effluent is not milky (2) the patient is hemodynamically stable with oxygen saturations at their baseline (3) the CXR shows lung expansion (4) there is no air leak on the digital drainage system, which we define by a leak of less than 20 mL/minute and only negative numbers on the pleural assessment test (9).

Patients have a planned discharge home on the morning of postoperative day 1 at 8 o'clock, as we have previously published (10, 11). Upon discharge, patients and their caregivers are given detailed instructions about communicating with the surgeon daily via text message with updates about how they are feeling in addition to pictures by text of their home pulse oximetry readings.

## Results

From May 2019 to April 2024, five patients underwent robotic distal tracheal resections on VV ECMO. Our cohort included three men aged 29, 74, and 37 years, and two women aged 11 and 40 years. Table 1 shows the patient demographics, presenting symptoms, and the location of the tumors. Four patients presented with symptoms, particularly hemoptysis in three patients and hypoxia/acute respiratory failure in one patient. The fifth patient was asymptomatic at the time of diagnosis; the tracheal tumor was found on a chest CT obtained for surveillance after a pulmonary resection for a stage 1A lung cancer he had years prior. All tumors were in the mid-to-distal trachea, approximately 1–6 cm from the carina. Four tumors were located 1–3 cm from the carina and one was in the mid-trachea approximately 5.5 cm from the carina.

**Table 1:** Demographics and patient characteristics.

Variable	All Patients *N* = 5
Age, years, median (range)	37 (11–74)
Sex, *n* (%)	
Female	2 (40%)
Male	3 (60%)
Location of tracheal tumor, *n* (%)	
1–3 cm above carina	4 (80%)
6 cm above carina	1 (20%)
Symptoms at time of diagnosis, *n* (%)	
Hemoptysis	3 (60%)
Acute respiratory failure	1 (20%)
Smoking history, *n* (%)	2 (40%)
FEV_1_%, median (range)	110 (78–110)
D_LCO_ %, median (range)	103 (82–110)
ECOG score, *n* (%)	
0	0
1	2 (40%)
2	3 (60%)
3	0
Diabetes mellitus, *n* (%)	0
Coronary artery disease, *n* (%)	0
COPD, *n* (%)	0
Hypertension, *n* (%)	1 (20%)
Hyperlipidemia, *n* (%)	0

FEV_1_: forced expiratory volume in 1 s; D_LCO_: diffusing capacity of the lungs for carbon monoxide; COPD: chronic obstructive pulmonary disease.

Table 2 shows the operative results. All operations were performed as described above. In each case we resected approximately 3–4 cm of trachea. Median operative time was 258 min (range 227–292 min). Median number of lymph nodes removed was 27 (range 23–29) and all patients had five N2 stations and one N1 station assessed. There was no evidence of an anastomotic leak on any intraoperative leak test. In all patients we were able to avoid systemic heparin, suprahyoid release maneuvers, a pericardial release maneuver, and a postoperative guardian chin stitch. All patients tolerated the operations well. There were no major complications. They were successfully decannulated from VV ECMO at the conclusion of the operation and extubated.

**Table 2:** Operative details and outcomes.

Variable	All Patients *N* = 5
Estimated blood loss in cc, median (range)	20 cc (10–40)
Operative time in minutes, median (range)	258 min (227–292)
R0 resection, *n* (%)	5/5 (100%)
Lymph nodes removed, median (range)	27 lymph nodes (23–32)
Length of trachea resected in mm, median (range)	30 mm (30–40)
Chest tube removal, *n* (%)	
In OR	2/5 (40%)
Within 4 h postoperatively	3/5 (60%)
Length of stay, median (days)	1 day in all patients
30-day readmission, *n* (%)	0
30-day mortality, *n* (%)	0
90-day mortality, *n* (%)	0
Final pathology, *n* (%)	
Squamous cell carcinoma	1 (20%)
Basaloid epithelial cancer	1 (20%)
Mucoepidermoid carcinoma	1 (20%)
Salivary gland type adenocarcinoma	2 (40%)
Recurrence of disease within follow-up to date, *n* (%)	0
Stricture within follow-up to date, *n* (%)	0
Postoperative complaints of hoarseness after 2 weeks, *n* (%)	2/5 (40%)
Postoperative complaints of cough after 4 weeks, *n* (%)	2/5 (40%)

There were no postoperative air leaks on the digital drainage systems. Chest tubes were removed within four hours postoperatively in three patients. In two patients (performed most recently in 2024), chest tubes were removed in the OR prior to extubating. Four patients were discharged home on the morning of POD1 by 8:00 AM and one patient left at 9:45 AM.

There was no minor or major morbidity and no 30 or 90-day mortality. There were no re-admissions. As shown in Table 2, two patients had mild hoarseness that resolved within 14 days. Two patients complained of persistent cough and underwent flexible bronchoscopy at six and twelve weeks postoperatively at their home institutions which showed intact, well-healing anastomosis. The final pathologies were squamous cell carcinoma, basaloid epithelial cancer, mucoepidermoid carcinoma, and salivary gland type adenocarcinoma in two. All were R0 resections and all had negative lymph nodes.

## Discussion

This is the first published series of tracheal resections for cancer using a robotic platform and VV ECMO since our first operation was performed in 2019 and presented at a national meeting. We have described the technical aspects and the safety and efficacy of robotic tracheal resection using VV ECMO. Since our first operation in 2019, Spaggiari et al. in 2023 (12) has also shown the safety and feasibility in one patient. Although their report differs in some minor ways (they used two chin sutures at the end of the operation, elected to use heparin and protamine, had an operative time of 420 min and a ten-day length of stay), there are more similarities than differences. Both have excellent and safe short-term outcomes, used a right-sided four-arm robotic approach and VV ECMO. In our series, all patients underwent R0 resections, none required adjuvant therapy, and none have had recurrence of cancer or stricture to date. However, follow-up remains brief and varied, from six years for our first patient to one year for our most recent patient. This series demonstrates feasibility and safety and the report by Spaggiari displays scalability.

The major limitation to this study is that it is a small series of only five patients and only one surgeon. The strength of this study is that all patients seen since 2019 with a primary mid-to-distal tracheal tumor have been offered this robotic approach on VV ECMO. There were no exclusion criteria for a robotic approach. It is not a selective series but rather a consecutive one without selection bias. In addition, the five patients were diverse in gender and age.

Performing distal tracheal resections for primary tracheal pathology robotically while on VV ECMO is safe and offers excellent short-term results. This technique offers all the oncological benefits of an open operation but adds the advantages of a minimally invasive surgical platform and recovery. In addition, the innovation of VV ECMO support, complemented with the benefits of robotic surgery provides excellent oxygenation and ventilation without the need for cross-table ventilation, which is often cumbersome and challenging to manage. This innovative, minimally invasive approach coupled with our enhanced recovery program of “chest tube-less” surgery and a 23-hour length of stay further improves patient experience and adds value to the healthcare system and the community so other patients can be offered care. This study of five patients shows it is safe and effective and is probably scalable to other surgeons and centers that are experienced in both robotic thoracic surgery and ECMO.

## Data Availability

The raw data supporting the conclusions of this article will be made available by the authors, without undue reservation.

## Supplementary material

Supplementary Video S1 The proximal trachea is dissected.

Supplementary Video S2 The distal trachea is dissected.

Supplementary Video S3 Intraoperative bronchoscopy is used along with the tile pro feature and infrared robotic camera to identify the proximal and distal extents of the tumor.

Supplementary Video S4 The distal and proximal tracheotomies are performed.

Supplementary Video S5 A tension-free, end-to-end anastomosis is performed using a running 3-0 barbed suture.

Supplementary Video S6 A leak test of the anastomosis is performed.
